# Draft Genome Sequence of *Bacillus licheniformis* VSD4, a Diesel Fuel–Degrading and Plant Growth–Promoting Phyllospheric Bacterium

**DOI:** 10.1128/genomeA.00027-17

**Published:** 2017-03-16

**Authors:** Vincent Stevens, Sofie Thijs, Breanne McAmmond, Tori Langill, Jonathan Van Hamme, Nele Weyens, Jaco Vangronsveld

**Affiliations:** a Centre for Environmental Sciences, Hasselt University, Diepenbeek, Belgium; b Department of Biological Sciences, Thompson Rivers University, Kamloops, British Columbia, Canada

## Abstract

We report here the 4.19-Mb draft genome sequence of *Bacillus licheniformis* VSD4, a Gram-positive bacterium of the *Bacillaceae* family, isolated from leaves of *Hedera helix* growing at a high-traffic city center in Belgium. Knowledge about its genome will help to evaluate its potential as an inoculant in phylloremediation applications.

## GENOME ANNOUNCEMENT

Various bacteria within the genus *Bacillus*, including members of *Bacillus licheniformis*, have previously been associated with diesel fuel degradation (1, 2) and plant growth promotion (3, 4). *B. licheniformis* VSD4 was isolated from the leaves of *Hedera helix* plants growing at a high-traffic city center in Belgium. *In vitro* analyses indicated that this bacterium is capable of utilizing diesel fuel as a carbon source and producing compounds related to plant growth promotion. Partial 16S rRNA gene sequence data showed that VSD4’s closest relative is *B. licheniformis* ATCC 14580 (GenBank accession no. CP000002).

RNA-free DNA was extracted from stationary-phase cells grown in LB medium using a PureLink genomic DNA minikit (Thermo Fisher Scientific, Waltham, MA, USA), prior to digesting and ligating sequencing adaptors/barcodes using an Ion Xpress Plus fragment library kit (Thermo Fisher Scientific). Processed DNA was size-selected (480 bp) on a 2% E-Gel SizeSelect agarose gel and purified using Agencourt AMPure XP beads (Beckman Coulter, Inc., Brea, CA, USA). The library dilution factor was determined using an Ion Universal library quantitation kit prior to amplification and enrichment with an Ion PGM Hi-Q Template OT2 400 kit on an Ion OneTouch 2 system. The enriched Ion Sphere Particles were quantified using an Ion Sphere quality control kit. Sequencing was performed on an Ion 316 Chip version 2 (Ion PGM system) with an Ion PGM Hi-Q View sequencing kit (Thermo Fisher Scientific).

In total, 428,739 reads (mean length, 289 bases) generated 124 Mb (114 Mb with ≥*Q*20) of data. Reads were assembled using MIRA version 4.0rc4 (5), trimmed into 270 contigs ≥500 bp, giving a consensus length of 4,188,588 bp at 39.4× coverage (largest contig, 471,073 bp; *N*_50_, 139,640 bp). The genome sequence of *B. licheniformis* ATCC 14580 was used as a reference to order the VSD4 contigs in Mauve (6, 7). Genome annotation was completed using RAST (8, 9) and NCBI’s PGAP (10). The genome of *B. licheniformis* VSD4 has a G+C content of 46.2% and includes 3,674 coding genes, 719 pseudogenes, 34 rRNAs (5S, 16S, 23S), 83 tRNAs, and five ncRNAs.

Enzymes related to the *Pseudomonas putida* GPo1 *n*-alkane degradation pathway (11) are encoded in *B. licheniformis* VSD4’s genome, including homologues of AlkH (aldehyde dehydrogenase), AlkK (acyl-CoA synthetase), AlkT (rubredoxin-NAD^+^ reductase), and AlkN (methyl-accepting chemotaxis protein). Further, homologous enzymes of *P. putida* G7’s naphthalene degradation pathway (12) were located, including all four subunits of naphthalene dioxygenase (NahA). Genes associated with plant growth–promoting characteristics were also present: indole-3-acetic acid, acetoin, and siderophore production. *B. licheniformis* VSD4 is being further evaluated as an inoculant to enhance phylloremediation of environments contaminated with diesel fuel–associated air pollutants.

### Accession number(s).

This whole-genome sequencing project has been deposited in GenBank under the accession number MLKN00000000. The version described in this paper is the second version, MLKN00000000.2.
